# Becoming Interprofessional: A Longitudinal Narrative Study of Professional and Interprofessional Identity Development Across Five Health Professions

**DOI:** 10.1177/10497323251333960

**Published:** 2025-04-26

**Authors:** Sheri Price, Lindsay Van Dam, Meaghan Sim, Cynthia Andrews, John H. V. Gilbert, Kelly Lackie, Natalie Kennie-Kaulbach, Evelyn D. Sutton, Hossein Khalili

**Affiliations:** 1 3688 School of Nursing, Dalhousie University, Halifax, NS, Canada; 2 Faculty of Health, 3688 Dalhousie University, Halifax, NS, Canada; 3 432234 IWK Health Centre, Halifax, NS, Canada; 4 Faculty of Dentistry, Dalhousie University, Haliax, NS, Canada; 5 8166 University of British Columbia, Vancouver, BC, Canada; 6 College of Pharmacy, 3688 Dalhousie University, Halifax, NS, Canada; 7 Faculty of Medicine, Dalhousie University, Haliax, NS, Canada; 8 School of Health Sciences, 606249 Winston-Salem State University, Winston-Salem, NC, USA; 9 Interprofessional Research Global (IPR.Global)

**Keywords:** inter**/**professional socialization, interprofessional education, collaborative practice, health professional students, dual identity, interprofessional identity, professional stereotyping

## Abstract

Interprofessional collaborative practice (IPC) occurs when health professions work collaboratively to improve quality of care and enhance patient outcomes. Yet myriad challenges to enacting collaborative practice exist. Interprofessional education for collaborative practice (IPECP) is foundational for promoting collaboration among health professions, yet there is a gap in understanding how students perceive their readiness for IPC and how early socialization experiences may contribute to developing a dual—uni-professional and interprofessional—identity. This study seeks to understand how new practitioners perceive and experience IPC upon entry to practice, and identify individual and systemic factors that facilitate and impede dual identity development. An interpretive, narrative methodology was used to understand the IPC and early professional practice experiences of 24 individuals from a longitudinal study of five health professions. Facilitators to interprofessional identity development included exposure to/working with interprofessional teams, settings, role models, and directly experiencing benefits of collaborative practice during patient care. Impediments include settings and situations where professional stereotyping and hierarchies were reinforced by the dominant uni-professional culture of work environments. Interprofessional socialization and identity development are contingent on exposure to interprofessional role models and settings. Healthcare professionals’ dual identity development begins in pre-licensure IPECP but is shaped by socialization experiences within practice. Healthcare institutions need to provide nourishing collaborative environments (time, settings, and contexts) that foster interprofessional collaboration and behaviors and empower dual identity formation. Post-licensure IPECP for healthcare professionals to continue to learn with, from, and about one another in practice is essential for collaborative interprofessional healthcare teams/systems.

## Introduction

In response to rapidly evolving public health needs and challenging events in the health of the population, there is an urgent need for health systems to adapt and integrate new models of care delivery inclusive of interprofessional teams and collaborative practice (Khalili et al., 2021; World Health Organization, 2010). Interprofessional practice, comprised of diverse health professions working collaboratively to provide patient care, has been consistently linked to higher quality and safer care delivery, improved patient outcomes, and higher rates of patient satisfaction with care received (Grant et al., 2024; Reeves et al., 2013; World Health Organization, 2010). Moreover, interprofessional teamwork has been reported to enhance the professional practice experience for care providers, promote workplace satisfaction, and act as a buffer to professional burnout and mental exhaustion that is concerningly on the rise among healthcare providers globally (Chang et al., 2019; Welp et al., 2016). Despite the overwhelming evidence in support of interprofessional teams and collaborative practice, myriad challenges persist in enacting interprofessional collaboration. Among diverse health professionals, the existent literature identifies turf wars and competing professional knowledge, hierarchy, and the uni-professional culture of healthcare systems as barriers, further inflaming historical issues of professional territorialism and hierarchical social positioning between health professions (Appelbaum et al., 2020; Kennie-Kaulbach et al., 2023; Khalili & Price, 2022; Noyes, 2022). Prevailing negative stereotypes and misunderstanding of the scopes of practice, roles, and responsibilities of other professions further impede interprofessional respect and collaboration (Ateah et al., 2011; Van Dam et al., 2024).

Evidence strongly supports interprofessional education for collaborative practice (IPECP) as foundational to health professions education to promote professional cohesion and collaboration, and combat preconceived professional biases (Khalili et al., 2013, 2022, 2024; Price et al., 2021a; 2021b). IPECP, by definition, includes processes, events, and experiences when members or students of two or more health professions “learn about, with and from each other, to improve collaboration and the quality of care and services” (Centre for Advancement of Interprofessional Education, 2016). Recent research has supported the implementation of IPECP early in the pre-licensure education of health professions to promote interprofessional socialization and identity development by enabling an early understanding of collaborative practice (Khalili & Price, 2022; Mukhalalati et al., 2024; Price et al., 2024; Stull et al., 2024).

Upon entry into a professional program, health professional students undergo formal processes of professional socialization, whereby they come to learn their professional role (Cruess et al., 2019; Sadeghi Avval Shahr et al., 2019). Professional socialization is considered a dynamic process happening simultaneously at the individual (psychological) and community (professional group) level (Jarvis-Selinger et al., 2012; Wenger-Trayner & Wenger-Trayner, 2015). Exposure to and frequent contact to a professional community enables processes of self-transformation, where students come to internalize the culture (attitudes, values, and beliefs) of their chosen profession—forming a professional identity (Sadeghi Avval Shahr et al., 2019). The formation of a professional identity is considered to be an essential component of students’ education and supports fulfilling expectations of their professional role in future practice (Cruess et al., 2019).

Exposure to IPECP during pre-licensure education supports students’ interprofessional socialization, which involves social contact with a broader professional group. Engaging socially with other professions in IPECP and exposure to collaborative scenarios is identified as a catalyst to developing an interprofessional identity (Reinders & Krijnen, 2023). An interprofessional identity is theorized not as a competing identity but as an extension of a professional identity, where individuals come to develop a sense of belonging and commitment to a broader, interprofessional community (Khalili et al., 2013; Reinders et al., 2018; Reinders & Krijnen, 2023). Social identity development and intergroup contact theory both support that when social contexts and conditions enable an individual to form affective ties with others and to identify with a (interprofessional) group, congruent social behaviors will form (Pettigrew, 1998; Tajfel & Turner, 1979). Behaviors such as enhanced collaboration, communication, and more effective teamworking have been characterized in the literature to be associated with the development of an interprofessional identity (Reinders et al., 2018, 2020; Reinders & Krijnen, 2023).

Fostering the development of a professional and interprofessional identity among students has therefore become a key priority of health professions programs to enhance collaboration and patient care in practice.

The concept of developing a dual identity (professional identity alongside an interprofessional identity) is described by Khalili and Price (2022) as the process of drawing upon the knowledge of one’s chosen profession and unifying this knowledge with understanding the expertise of another, as facilitated through collaboration and interprofessional teamwork. Developing a dual identity not only promotes stronger team functioning but also fosters an increased sense of personal accountability to one’s fellow interprofessional team members (Khalili & Orchard, 2020; Khalili & Price, 2022; Reinders & Krijnen, 2023). Processes for interprofessional identity development are not well described in the literature, yet development remains a critical component for successful collaboration between health professions in practice. The introduction of IPECP experiences early in health professions students’ educational experience has been challenged in the literature, citing limitations for developing an interprofessional identity when students are still coming to understand and develop their own identity within their new professional role (Odole et al., 2019; Shelvey et al., 2016; Whiting et al., 2016). More recent evidence however has been found to support early IPECP for learning interprofessional communication skills and beginning processes of developing a dual identity (Price et al., 2021, 2024; Stull et al., 2024). To meaningfully target students’ dual identity development, there is support for offering IPECP experiences within and beyond the first year, and experiences integrated throughout the curricula of students’ formal program years (Grace, 2021).

Emerging evidence from longitudinal analyses of health professions students’ IPECP experiences has shown its impact on developing values, attitudes, and behaviors conducive to collaborative interprofessional practice upon program completion (Gunaldo et al., 2021; Kavanaugh et al., 2023; Price et al., 2024; Van Dam et al., 2024). While there is compelling evidence to suggest that when students engage in longitudinal IPECP they are better prepared for collaborative practice upon graduation and entry to practice, there are very few studies that have followed graduates into professional practice (R. Tong et al., 2022). As a result, there is little evidence available to confirm and support the design of current IPECP models used in health education. There is a noted gap in our understanding of how students perceive their readiness for interprofessional teamwork and how their early practice experiences contribute to, or challenge, the development of dual identity, with implications for how they practice interprofessionally. This information is critical to inform and enhance the IPECP knowledge about the healthcare environments that foster interprofessional learning for students and graduates in practice settings. Without longitudinal analyses of graduates within their early years of practice, health professional programs are limited in their understanding of the effectiveness of current IPECP approaches. Moreover, there is a lack of evidence to inform where areas for strategic development of IPECP must be implemented to better reflect the real-world experiences of new practitioners.

This study aims to address this gap and builds on an existing study that longitudinally followed students from five health programs. Student experiences and perceptions of IPECP during their early years of pre-licensure training for professional and interprofessional identity development and collaborative practice readiness have been published elsewhere (Price et al., 2021a; 2021b; Price et al., 2024). This study follows the same cohort of students from five health professions into their first few years of practice. The purpose of this research is to explore how new healthcare professionals perceive and experience interprofessional collaboration upon entry to professional practice and to better understand the individual, interpersonal, and systemic challenges and facilitators within their practice environments for developing/advancing dual identity and working effectively within interprofessional teams.

## Methodology/Methods

An interpretive, narrative methodology was employed in this study to better understand the interprofessional practice experiences, and professional practice contributions to self-identity development for healthcare practitioners in their early years of professional practice (Polkinghorne, 1997). This narrative inquiry was guided by philosophical and theoretical principles of interpretivism, hermeneutics, and narrative identity theory (Polkinghorne, 1995, 1997; Ricoeur, 1991, 1997). These principles are complementary and intersecting; when applied together, they contribute to the development of a coherent story that is purposeful and consciously accounts for participants’ personal experiences. Narrative approaches require in-depth interaction with participants’ narratives overtime to develop understanding of events, experiences and their contributions to one’s self-identity. An attentiveness to influential professional practice events and experiences was maintained throughout this study and during the analysis, in addition to a sensitivity to the ways in which individuals organize and recount influential events within their personal narratives which is necessary to derive meaning.

### Participants and Study Setting

A convenience sample of 24 individuals from a longitudinal study of health professional students was used in this study. Students were followed from pre-program entry to their early experiences of professional practice. Earlier findings on students’ pre-licensure IPECP experiences have been previously published (Price et al., 2021a; 2021b; Price et al., 2024). Individuals who had consented to participate in interviews during their pre-licensure education (*n* = 49) were contacted via email with an invitation to participate in a final follow-up interview about their early experiences within professional and interprofessional practice. The 24 individuals in this study represent the following health professions: dentistry (*n* = 2), pharmacy (*n* = 3), physiotherapy (*n* = 6), medicine (*n* = 8), and nursing (*n* = 5). All individuals were graduates of a large public university in Eastern Canada and began their studies in the Fall of 2015. Graduates of the physiotherapy program were contacted 4 years into their professional practice (2-year pre-licensure program) and all other professions 2 years into professional practice (4-year pre-licensure program). All individuals, as part of their program curricula during pre-licensure education participated in, and completed, mandatory IPECP. Details of students’ IPECP curricula have been previously reported elsewhere (Price et al., 2021b). These five health professions were initially recruited for a longitudinal study on IPECP and professional identity as they represent the largest cohorts of health professions students at the institution where they were enrolled and all represent healthcare providers with evolving and overlapping scopes of practice, roles, and responsibilities. Further, these professions are identified as those that may collaborate, or share collaborative practice spaces, in future practice. Apart from identifying which professional program they had completed, no additional demographic information was collected from participants. All participants provided informed consent to participate in this study. Approval for this study was obtained from the Health Sciences Research Ethics Board at Dalhousie University (REB#: 2014-3416).

### Data Collection

Participants of the longitudinal pre-licensure study, now early into their professional practice, were invited to participate in a final follow-up interview and those interested in participating were reconsented by a research assistant. Consistent with narrative inquiry, one-on-one semi-structured interviews were conducted with participants over several months in 2021. Interviews were conducted by the research coordinator (RC), a postdoctoral fellow who had previous experience interviewing the same cohort of participants during their pre-licensure education. A flexible, semi-structured interview guide (Price et al., 2024) was used to gently direct and prompt discussion only as needed to maintain closeness with the topic. Interview questions pertained to the practice experiences of new healthcare professionals and their perceptions and experiences of interprofessional socialization and collaborative practice. Participants were also prompted to reflect on their experiences in professional practice to date and how they conceptualize their role and identity as professionals. All interviews were conducted virtually using video-conferencing software (Microsoft Teams®). All interviews were video-recorded, transcribed verbatim, and reviewed for accuracy. Participants were de-identified at the time of transcription by the RC and assigned a numeric code to organize the data. All participants were assigned a pseudonym for use of their quotes in this manuscript to maintain confidentiality.

### Data Analysis

An interpretive approach guided by Polkinghorne’s (1995) theory of narrative configurement and emplotment and Beiter’s (2007) narrative analysis framework was applied to transcribed participant data (Beiter, 2007; Polkinghorne, 1995). Concordance with COREQ guidelines for qualitative research was maintained throughout while dually maintaining thoughtfulness to the hermeneutic origins of narrative inquiry (A. Tong et al., 2007). A sub-set of transcripts was independently thematically characterized by two members of the research team (SP and LVD) for identification of plots, subplots, characters, settings, and tensive points. The two authors met to discuss themes identified and reconcile any conflicting interpretations. Following investigator triangulation and consensus on initial themes identified, all interviews were subject to simultaneous analysis. Following analysis of all transcripts, emergent themes were synthesized into a meaningful hermeneutic narrative account of participants’ stories of their professional practice experience, socialization within practice settings, and their perceptions of interprofessionalism. All emergent themes and harmonized narratives were reviewed, discussed, and validated by the wider research team. The confirmation of trustworthiness in the data as representative of participants’ narratives from research team members, representing diverse professional health educator and researcher backgrounds, promotes the rigor and authenticity of the narrative presented.

### Investigator Positionality

All authors have backgrounds as practicing health professionals, health educators, and researchers in Canada and the United States. Among the authors, there is professional representation from all five health professions included in the research. There is extensive experience in qualitative research methods among the authors listed and all have intimate knowledge of the health professions represented in this study and insight into how these professions are trained for, and may participate in, interprofessional collaboration in their everyday practice. The lead author and principal investigator, SP, is a registered nurse and professor in the School of Nursing at the institution of study. They are also an expert in the field of interprofessional education and interprofessional socialization within the health professions and widely published on the topic.

## Results

### Becoming Interprofessional

Across the narratives, participants described their interprofessional identity development as an evolution over time that is centered on exposure to collaborative practice. Having the opportunity to work in interprofessional healthcare teams and experiencing the benefits of collaborative practice were narrated as an extension of participants’ formal education. Participants’ embodiment of IPECP skills, attitudes, behaviors, and competencies in practice settings contributed to their ability to develop an interprofessional identity. Within the participant narratives, characters, settings, and context were found to be central to the ability to develop an interprofessional identity and continued IPECP (Table 1).

**Table 1.** Themes, Context, and Participant Quotes.

Theme	Context	Quote
Becoming interprofessional	Experiencing interprofessionalism	“I would meet these people socially, whether in the hospital or outside the hospital … and it made it so much easier when I would see them in emerg or somewhere else. It’s like we already knew each other and got on well. And then to ask for their help and ask their opinion, it was so sweatless. I didn’t feel intimidated at all. And so the social elements really allowed for some of the more learning opportunities.” (*Medicine graduate*)
		“Just the day-to-day, like you really are collaborating with so many different disciplines constantly…the more exposure in the workplace honestly was what helped [to learn interprofessionalism].” (*Nursing graduate*)
		“Until you actually see [other team members] work in their environment...you just don’t really understand how it all comes together until you see it in progress.” (*Medicine graduate*)
Impediments to interprofessionalism	Professional stereotypes and hierarchies	“Talking to doctors- everyone finds it so intimidating, and they all get nervous when they talk to a doctor. And I was definitely nervous my first year [in practice] having to like page a doctor and explain and talk and interact.” (*Nursing graduate*)
		“[I wish] there was more willingness to allow people to just like take a step into your scope of practice and ask the questions they want to without fear of backlash.” (*Pharmacy graduate*)
	Separate spaces and practice culture	“There would never be a setting where I would happen to be eating lunch with a pharmacist because, I don’t even know what space that would be in. I don’t know where they go, but they go somewhere that is not the [medical] resident lounge. And I go to the resident lounge.” (*Medicine graduate*)

#### Experiencing Interprofessionalism

Clinical settings that were interprofessional, with diverse groups of health professionals working collaboratively, were central to participants’ understanding of collaborative practice. The most impactful learning experiences centered on witnessing respectful communication and role valuing among interprofessional team members. Exposure to interprofessional settings and teams where positive communication and clear role understanding were role-modelled was a prominent plotline within participants’ narratives of becoming collaborative professionals. Interprofessional rounds were an example of an experience that showcased respectful communication and role clarity and enabled dual identity development. For participants like Kareem, a medical resident working in the hospital emergency room setting, observing supportive and respectful communication and well-defined roles among team members was influential in understanding interprofessional practice and the importance of collaboration in patient care:

The way those teams work together and how everyone is on the same level in those situations .. the roles are well defined. And there’s a courtesy and a positive reinforcement. People are like, “Great! You’re doing a great job!” That sort of stuff. And also just, very to-the-point communication. It’s cool to see that play out.

Evident in Kareem’s quote was also an acknowledgement of respect for profession-specific knowledge/skill and the absence of hierarchy in patient care delivery where everyone was “on the same level.” Opportunities to observe and participate in interprofessional team rounds and debriefs following patient care events were narrated as important experiences for participants to see positive and effective communication among interprofessional team members. When collaborative communication and leadership were evident during patient care planning and delivery, participants had an enhanced appreciation of the role and contribution of each member to team functioning.

Experienced practitioners who were strong collaborators served as role models for effective communication and collaboration. Zoey, a physiotherapist new to working on the hospital unit, described being paired with an “absolutely phenomenal” rehab assistant during her first shifts in which she was exposed to respectful and clear interprofessional communication and a high-functioning team. As Zoey described, exposure to collaborative role models in practice promoted a team mentality which enabled her to feel “melded together” with her colleagues. Professional peers who role-modelled respect, effective communication, and collaborative teamwork demonstrated the benefits of working together, rather than uni-professionally, to the provision of patient care.

The context and timing of meaningful experiences are essential components of narrative emplotment, and it is important to note that the moments in which participants narrate the greatest understanding of interprofessional practice often centered on the provision of direct patient care. In terms of timing, many of the instances that participants witnessed respectful communication and teamwork were in times of heightened stress, urgency, or complexity—such as patient codes or trauma situations where the knowledge, expertise, and skill of diverse team members were essential to positive patient outcomes. Participants like Jiya, a registered nurse, described the emergency department as a setting where interprofessional collaboration and respectful communication was “definitely a major part of the job.” For this participant, the urgency of delivering effective patient care was perceived to optimize interprofessional collaboration. Moreover, as Jiya reinforced, the emergency room setting was inherently interprofessional—where collective identity was “team.” As Jiya shared,

In Emerg, I mean my goodness, like it doesn’t really matter what profession you are in, when we are in dire straits, it would be—“do you know how to do this? Okay, great- go for it!”

Outside of patient care scenarios, informal socialization between interprofessional team members provided an opportunity for the participants to get to know other professionals as people, which appeared to enhance collegiality and respect. Informal socialization could consist of casual interactions between team members on the unit in between patient care, during work breaks, or gatherings outside of the workplace. As the narratives reveal, the importance of getting to know interprofessional team members as individuals promoted team bonding while also enhancing interprofessional collaboration.

Participants, as novice practitioners, identified that formal and informal interprofessional socialization created an environment in which they felt more comfortable asking questions, which thereby enhanced role clarity. An environment in which there is collegiality was narrated as a setting that enhanced interprofessional collaboration. Moreover, many participants described that these collaborative environments created a feeling of psychological safety in terms of willingness to express vulnerability and ask for help, which promotes a sense of belonging to the interprofessional team. As Maria, a new family medicine physician, reflected on her residency experience in the hospital,

I would meet these people socially, whether in the hospital or outside the hospital … and it made it so much easier when I would see them in emerg or somewhere else. It’s like we already knew each other and got on well. And then to ask for their help and ask their opinion, it was so sweatless. I didn’t feel intimidated at all. And so the social elements really allowed for some of the more learning opportunities.

Alex, now a family medicine physician, narrated the importance of reciprocity in seeking support from one another in practice. An environment in which you could ask each other’s opinions was narrated as an example of interprofessional collaboration. During his locum experience, Alex shared the impact of being able to seek help from others, while also being sought out for his advice:

Everyone’s really nice, they’re very approachable, very willing to give you some mentorship. Like when you ask, “oh, what would you do with this patient?” [or], “what do you think of my plan for this problem?” and they [other professionals] would tell me their thoughts. And they also asked my opinion of things, which I thought was really nice.

Participant narratives revealed that conditions which enabled and promoted collaboration in practice were pivotal to both their professional and interprofessional identity, or dual identity, development. The interprofessional working environment was described as its own unique “ecosystem” that is conducive to learning and becoming interprofessional. As Jiya (registered nurse) described,

Just the day-to-day, like you really are collaborating with so many different disciplines constantly … the more exposure in the workplace honestly was what helped [to learn interprofessionalism].

For participants like Joni, a nurse on a general surgery unit, observing how other members of the team embodied and demonstrated collaboration was fundamental to learning how to be a team member and practice collaboratively.

I always noticed that everyone’s helping the other person, everyone’s a team player. Like if you get a post-op coming right at shift change, everyone would grab something to like help you do the whole entire process.

Participants who worked in practice settings where routine exchanges between different professionals were limited described the importance of recognizing the benefits of collaborative practice to patient outcomes. Even in the absence of working alongside other health professionals in practice, understanding and valuing the benefits of collaboration enabled professionals to feel safe in reaching out to others for advice/support. As Salme, a dentist working in private practice, shared:

At first I would be a little nervous to reach out to [other professionals] thinking, like, I should know this, or maybe I don’t know enough to ask these questions, or maybe I wouldn’t need to reach out. But ultimately, whenever I did, I always felt better, and got a good quality response from every single person that I had reached out to. And it definitely made treatment better for the patient in the end.

Many participants learned the value of collaborative practice by witnessing the enhanced patient care delivery and outcomes achieved when professions worked together. Developing an interprofessional identity was influenced by exposure to the knowledge and skills of other professions in the provision of patient care. Through collaborative practice experiences, participants learned more about their peers and different scopes of practice. Over time, participants also narrated an awareness of the limitations of their profession-specific knowledge and skills, alongside a growing respect for the expertise of other health professionals.

I think the more you see, the more you realize how broad people’s scopes are. And that it involves so much more than the one or two lines you could probably say about that profession initially. (Maria, family medicine physician)

The narratives also revealed that understanding others’ scopes of practice required real-life exposure to teams and collaborative practice to fully understand the complementary nature of interprofessional practice. As Kai, a medical resident, shared:

Until you actually see [other team members] work in their environment ... you just don’t really understand how it all comes together until you see it in progress.

Another key element in the development of participants’ interprofessional identity was the understanding that they were all working together toward a shared goal of enhancing patient care. Complex patient care scenarios, where participants became aware of the limitations of their own knowledge and skills, were significant events for learning to rely on others in the provision of patient care. An evolved understanding of the benefits of collaborative practice was enhanced by repeated exposure to interprofessional collaboration. For Danielle, an internal medicine resident, her experiences in the hospital’s clinical teaching unit provided many opportunities to observe other professions delivering comprehensive patient care which increased her understanding of, and confidence in, other team members’ knowledge, skill, and expertise:

I just completely trust [other health professionals] judgement because … we’re all kind of working on the same team towards the same goal.

For Abdul (dentist), being exposed to collaborative practice post-graduation enabled his interprofessional identity development—seeing himself as more than just a dentist but a health professional team member—something that eluded him in undergraduate education.

You’re not a one-person army. You’re not going to do this alone .[...] It’s a team effort. I cannot emphasize this enough. And believe me, I didn’t understand the true meaning behind that while I was student, until now.

### Impediments to Interprofessionalism

While exposure to interprofessional collaborative teamwork and environments was identified to provide impactful and meaningful opportunities for participants to develop a dual identity, impediments to interprofessional collaboration were also evident within the narratives. Participants described an unpreparedness among new and experienced healthcare professionals for collaborative practice, including a lack of knowledge of how collaboration is enabled. Participants commonly described settings and situations where professional stereotyping and perceived hierarchies between the health professions created barriers to collaborative practice. Profession-specific stereotypes and devaluation of others’ professional roles and contributions to the interprofessional team were embedded in the culture of some healthcare practice settings. Professional misunderstanding, uncooperative behaviors, and disrespectful communication among healthcare professionals threatened collaborative practice. Working within non-collegial settings were narrated as contexts that bred competition rather than collaboration. Patient care scenarios where hierarchies prevailed, especially in the context of who was the identified team “leader,” created tensions and impeded collaborative practice. The role of authority, or leader, was narrated to be an accepted hierarchy and social ordering of the professions that remains dominant within the culture of certain practice settings. Alice, a physiotherapist working in a private clinic, described being socialized to professional hierarchies throughout her education and practice experiences. For this participant and others, hierarchical positioning of professions was commonly related to the professional prestige associated with the level of degree held. Evident in Alice’s experience is the challenge hierarchies pose to collaborative patient care:

There’s that hierarchy of health professionals. The surgeon’s at the top, and doctors. And the chiropractors seem to fall above physio. Which yes, they’ve got a doctorate so it makes sense.. but I find it hard to work collaboratively at times with a couple of those health professionals and trying to work cohesively with them on clients.

Like Alice, many participants confirmed an acceptance of medical physicians as holding the role of leader and the ultimate decision-making authority within healthcare. The positioning of medicine as leader was reinforced in the daily attitudes and behaviors of other health professionals in team-based care settings. The emplotment of the participants’ stories revealed that the physician is often depicted as the central/main character within healthcare teams, and other health professionals are positioned as supporting characters. As Jiya (registered nurse) described her practice setting in the hospital,

It’s very much like, “Oh Dr. So-and-so needs this”. We need to drop everything and do this for them … it’s very doctor-focused.

For new non-physician professionals, the reinforcement of their “supporting character” role by fellow team members impeded their ability to fully realize their professional roles within practice. Moreover, professional and social hierarchies were described by participants as fostering sentiments of intimidation between professions to the detriment of team collaboration and functioning during patient care. Participants described a hesitation to engage in communication with physicians during complex patient care experiences when they needed to consult or seek clarity on patient care orders. As described by Joni (registered nurse),

Talking to doctors- everyone finds it so intimidating, and they all get nervous when they talk to a doctor. And I was definitely nervous my first year [in practice] having to like page a doctor and explain and talk and interact.

The prevalence of professional hierarchies was also narrated to influence how professionals spoke with, and about, each other. Deprecating and disrespectful language was identified as a pattern where some professionals competed with one another and sought to legitimize their professions’ status and knowledge in the “pecking order” of the health professions. The experience of witnessing or working within a dysfunctional team was also narrated as a deterrent for participants in choosing certain settings for employment. As Christina shared, the competition between professions and psychological toll of needing to “defend” her professional knowledge within an acute care hospital setting influenced her decision to seek a uni-professional practice with other physiotherapists.

There’s definitely a thing within health care ... of putting other professions down to build your profession up. I definitely felt like my guard had to be up versus like trying to have more of an open approach and being willing to learn. Like I almost had to defend myself and what I was doing.

Attention to setting is a key feature of narrative research and the narratives reveal that physically divided or siloed spaces also hindered collaborative practice. Profession-designated break rooms and consultant offices with “closed doors” were narrated as settings that reinforced segregation and disconnection between professionals that impeded both collaboration and informal socialization between team members. Describing the design and location of spaces in her pediatric hospital practice, Laura (medicine) confided,

There would never be a setting where I would happen to be eating lunch with a pharmacist because, I don’t even know what space that would be in. I don’t know where they go, but they go somewhere that is not the [medical] resident lounge. And I go to the resident lounge.

Participant narratives also revealed how deeply engrained the acceptance of professional divide was within the social culture and professional practice standards of certain healthcare settings. For some participants, learning to navigate professional hierarchies and socially accepted behaviors on top of learning their professional role and patient care responsibilities contributed to negative perceptions of interprofessional healthcare practice. Lia, a registered nurse, narrated challenges in learning how to obey the “rules” of healthcare practice and settings.

There’s a lot of unspoken stuff that I think maybe we’re supposed to pick up. Maybe other people do pick it up. Sometimes it just feels like I’ve walked into a room, and there was a rule book given out but it wasn’t given to me. Sometimes I find that just as exhausting as the job itself.

The narratives of new professionals in practice reveal the need to address hierarchies and uni-professional practices within current healthcare settings that are impeding collaborative practice and the development of interprofessional teams. Many participants relayed that they believed their IPECP experiences in school provided “foundational” knowledge of other healthcare professions and roles which facilitated interprofessional identity development. However, they also identified a need for exposure to “real-world” experiences of collaborative practice to empower them as change agents to promote a collaboration, nurture an interprofessional identity, and develop the competencies conducive to collaborative practice.

## Discussion

The early practice experiences of participants in our study identify and confirm the critical role of exposure to interprofessional socialization for addressing individual, interpersonal, and systemic challenges for collaborative practice. Formal and informal socialization opportunities between professionals in healthcare settings are narrated by participants as foundational for learning professional roles and collaborative teamwork. In addition, they were identified to support developing a sense of belonging to a team, which is consistent with intergroup contact and social/interprofessional identity development theories (Pettigrew, 1998; Reinders et al., 2020; Stets & Burke, 2000). Exposure to settings where collaborative behaviors and team leadership are positively role-modelled by others was identified to be essential for developing an interprofessional identity and empowering collaborative capacity in new healthcare professionals. A significant finding in our study was that interprofessional practice entailed more than just having diverse groups of health professionals working in the same setting which is multiprofessional practice versus interprofessional collaboration (IPC). Narratives identified true interprofessional practice settings as spaces where respectful communication and role valuing were consistently modelled by other team members. Important facilitators for the creation of interprofessional settings and teams were identified as settings that provided opportunities for cooperative, collaborative, and receptive interactions between professionals. Exposure to respectful and supportive interprofessional communication with others created space for cultivating collaborative, rather than uni-professional, practice culture. For new professionals, interprofessional identity development is found to be best supported within settings where mutual respect, collaborative leadership, and role valuing can be experienced and developed in the collaborative practice education curricula.

The findings of this study support the interprofessional socialization (IPS) framework proposed by Khalili et al. (2013). Participants’ experiences in working alongside, and in collaboration with, other professionals in practice highlight the iterative nature of the three stages of IPS toward the development of dual identity. The three stages include: breaking down profession-specific barriers to collaboration; engaging in collaborative experiences and learning to work effectively as a team; and developing a sense of belonging to both one’s profession and the broader interprofessional community. Our study also provides further support and attention to the Canadian Interprofessional Health Collaborative’s (CIHC) Framework for Advancing Collaboration (CIHC, 2024). Within the framework, four targeted domains (role clarification and negotiation, team functioning, conflict resolution, and collaborative leadership) for interprofessional education and competency development among healthcare students and practitioners are identified to support and enable interprofessional collaborative practice for patient-centered care (CIHC, 2024). Facilitators for participants’ learning collaborative skills in our study, such as exposure to healthcare settings where role modelling of collaborative behaviors, attitudes, and role valuing are experienced, are directly linked to positive competency development across these domains. In addition, our findings are consistent with Reinders et al.’s (2020) extended professional identity theory (EPIT) which emphasizes the significance of observational learning for interprofessional relationships and skills development. Exemplars within our findings included participant descriptions of seeing and experiencing “helping” behaviors and “team player” attitudes from others in practice. When reciprocal behaviors between health professionals are observed, particularly in settings and situations where social behaviors are linked to positive performance, these behaviors become internalized as interprofessional behaviors (Reinders et al., 2018, 2020). Identified from the narratives was that several participants learned and came to develop congruent interprofessional behaviors in response to collaborative experiences and exposure in practice. Behaviors are identified in the social psychology and identity development literature to follow from processes of group identification, signalling to continued interprofessional identity development among these participants within professional practice (Reinders et al., 2018; Stets & Burke, 2000; Tajfel & Turner, 1979).

Our study identifies that the development of collaborative behaviors among professionals on entry to practice is positively influenced by early exposure to IPECP during their pre-licensure education. As students, participants in this study engaged in curriculum-embedded IPECP experiences that were guided by and developed according to the CIHC framework. IPECP experiences for students centered on interprofessional communication and learning professional roles were part of their program curricula (Price, et al., 2021a; 2021b; Price et al., 2024). Their experiences support the effectiveness of these early events for developing understanding and skills that enabled collaboration with other professionals. Participants in our study were found to model learned IPECP competencies in practice and continued to develop/advance role clarification, team functioning, and dual identity formation post-graduation.

Recent studies support the positive implications of early interprofessional socialization between healthcare professions beginning in pre-licensure education for interprofessional conflict resolution and beginning processes of resolving misunderstanding and stereotyping between professions (Price, et al., 2021a; 2021b; Price et al., 2024). The narratives of participants in our study support and reinforce a need for the integration of interprofessional experiences throughout students’ program curricula to enhance respectful communication, role valuing, team functioning, and other collaborative behaviors. Experts in the field of interprofessional behaviors identify that learned communication skills are a challenge for individuals to maintain long-term unless used and modelled consistently within daily practice (Ammentorp et al., 2022; Kruijver et al., 2000; Wolderslund et al., 2021). Our study underscores the risk that exposure to disrespectful communication and behaviors among healthcare professionals within practice settings poses for sustained development of collaborative skills and attitudes long-term. Our findings suggest there is a need for integrated IPECP curriculum in the health professions centered on empowering and developing new professionals to be role models for collaborative practice.

The early development of collaborative competencies by new professionals in our study ardently supports the potential of healthcare professionals to assume roles as effective change agents for interprofessional culture and practice within healthcare settings. Participants who were unable to break through barriers of uni-professional healthcare practice because of the current culture and contexts of healthcare settings described being unable to work collaboratively within the healthcare team. Participants also described exemplary scenarios, settings, and interactions with other professionals in practice where disrespectful behaviors and negative attitudes toward others were commonly encountered. Not only were these instances identified as barriers to effective collaboration, but they were found to impede new practitioners’ ability to continue forming a dual identity post-licensure. Consistent and frequent social contact between professionals is needed to form affective ties with others, establish a group identity, and develop congruent social behaviors (Pettigrew, 1998; Reinders & Krijnen, 2023).

For health professionals with previous IPECP experience, such as our participants, our study suggests that continued interprofessional identity development in practice can be obstructed when uni-professional cultures and attitudes dominate. Whether or not other professionals in practice were exposed to IPECP as students is unknown. However fewer opportunities to experience positive social contact or to be exposed to IPECP post-licensure and within practice settings may be contributing factors to the uni-professional versus interprofessional behaviors and identities noted in the narratives.

Our findings suggest that supports are lacking across many healthcare settings for professionals to work collaboratively, and to have opportunities to form and continue cultivating collaborative skills and behaviors. One recommendation from our findings is that health systems, institutions, and managers commit to addressing deficits for effective healthcare teams and delivery of collaborative patient-centered care by implementing and providing longitudinal and sustained IPECP and continuing education (CE) professional development opportunities. Interprofessional learning is recognized as a form of cumulative learning that must happen on a continuum; therefore, post-licensure IPECP and CE opportunities are needed that enable ongoing competency development for collaborative practice. This need is also consistent with interprofessional socialization and identity development theory which recognizes professional and interprofessional identity development as evolving psychological processes that happen over time and experiences (Reinders et al., 2018; Reinders & Krijnen, 2023). Consistent IPECP during pre-licensure education and in extension to post-licensure healthcare settings is a coveted strategy to accelerate the integration and adoption of CIHC collaborative competencies into real-world practice settings and in nurturing dual identity development among practicing professionals (CIHC, 2024).

Our study also highlights a need for shared, interprofessional physical spaces and settings for health professionals to engage in IPECP. Shared spaces and settings where participants had opportunities to discuss patient care scenarios with other team members were described as valuable opportunities for role valuing and learning their uni-professional and interprofessional roles. Within students’ IPECP experiences, including in healthcare practice settings, ongoing opportunities and spaces for formal and informal socialization and collaboration among professions are identified in recent studies to promote understanding of team functioning and to help generate affective ties between professions (Brewer et al., 2017; Mukhalalati et al., 2024; Stull et al., 2024).

Exposure to patient care rounds and debriefing sessions with all members of the interprofessional team were cited as exemplar settings to learn respectful communication and collaborative behaviors. Participant narratives identified interprofessional rounds as one setting that can positively promote rapport, trust, and understanding among team members. Restructuring interprofessional rounds where different professions lead group discussion on patient care events is a method by which healthcare settings may elicit improved understanding of professional scopes and knowledge, thus improving role valuing and team functioning. Interprofessional rounds and group discussion were found to enable collaboration and were narrated as events that provided opportunities to see collaborative leadership positively role-modelled by experienced practitioners. IPECP for students in health education programs that facilitates shadowing experiences of exemplar real-world interprofessional rounds is a strong recommendation from our findings to support modelling of collaborative behaviors early in their professional development. Further, healthcare organizations should focus on allocating resources that support increasing the frequency of rounding/debriefing events that can continue to build collaborative skills in practice. Integrating group socialization opportunities within professional practice settings can promote regular exposure to collaborative behaviors. In addition, social opportunities can support ongoing development of interprofessional understanding, role valuing, and respectful communication across individuals and teams (Coggins et al., 2021; Van Dam et al., 2024; Velásquez et al., 2022).

Exposure to collaborative patient care experiences was also identified as an important facilitator for participants’ interprofessional identity development. Simulation-enhanced IPECP and case-based learning experiences are found to be effective educational models for learning collaborative team-based skills and opportunities for team debriefing and role defining among team members (Khalili & Orchard, 2020; Maben et al., 2023). Collaborative IPECP models are employed within pre-licensure education in the health professions and can be adopted in continuing professional development and education opportunities for practicing professionals. Healthcare institutions should look to establish partnerships with professional associations in sponsoring interprofessional continuing professional development and education events. Interprofessional events can also promote socialization and understanding among professions that translate to collaborative leadership and effective teamwork in patient care delivery.

This need is prudent as the practice experiences of participants in our study highlight a failure of contemporary health systems to adequately assess for, and address, disrespectful behaviors through IPECP resources and training as well as collaborative policy development. Participants who were exposed to disrespectful behaviors from other professional team members confirmed feeling intimidated by their colleagues, which impeded socialization and collaboration. Disrespectful and non-collegial interactions between professionals are considered root causes of ineffective healthcare teams and are further linked to reported increases in adverse events and compromised patient care (Ammentorp et al., 2022; Grissinger, 2017; Guo et al., 2022). A lack of organizational attention to this issue creates broad systemic challenges for addressing and deconstructing uni-professional attitudes and culture, threatening effective collaboration between professionals in patient care. Moreover, a lack of organizational policy and IPECP-centric resources within practice settings are identified as major contributors to the proliferation of contentious workplace culture and dysfunctional healthcare teams (American Medical Association, 2021).

Our findings also identify barriers to interprofessional collaboration, as the existence of a “hidden curriculum” in healthcare settings that is implicit and unspoken but translated through individuals’ attitudes and behaviors (Hafferty & O’Donnell, 2015). Unwritten rules, norms, and values within these settings are found to teach and reinforce professional divide, contributing to cultures of disrespect. As identified in Khalili et al.’s (2013) framework, “breaking down barriers” is the first step toward dual identity development, and the findings in our study highlight a need to deconstruct professional stereotyping, turf wars, and hierarchy between professions that are significant barriers against learning and working with one another in healthcare settings (Khalili et al., 2013). Participant narratives commonly described disrespectful communication, lack of collaborative leadership, and competitive attitudes between professions during patient care situations. Notably, disrespectful interactions were narrated as socially engrained behaviors within practice, often occurring without intervention or organizational repercussion. Healthcare settings are identified in the literature as workplace environments at high risk for the proliferation of hostile and harmful behaviors between members (American Medical Association, 2021) Stressors such as challenging patient care scenarios and inadequate and failing health systems structures are cited as contributors to harmful workplace culture (Grissinger, 2017; Guo et al., 2022; Maben et al., 2023).

Mobilizing organizational strategies such as increased integration of IPECP opportunities that support interprofessional competency development and that bring awareness to disrespectful behaviors and conduct is needed to address barriers to interprofessional collaboration and effective team functioning. Moreover, the narratives of participants in our study reveal that physical barriers to socialization exist in the design and structure of contemporary healthcare practice settings. Positive interaction between healthcare professionals that supports interprofessional understanding, role valuing, and collaborative team/group identity formation is challenged by practice settings that physically enable professional divide. The ability for practitioners to mobilize learned IPC competencies and be change agents for interprofessional healthcare practice will be partially contingent on addressing these identified barriers. The authors strongly support the need for healthcare settings to initiate policy formation/reformation and IPECP opportunities for health professionals to disrupt challenges identified in this study for developing collaborative teams and an interprofessional identity.

### Strengths and Limitations

This 8-year longitudinal study provided an in-depth understanding of interprofessional socialization and identity development from pre-program entry to 2 to 4 years into practice. This study is unique in both the qualitative approach and longitudinal design both of which provide an in-depth understanding of dual identity development and IPC that currently does not exist in the published literature. The invitation to participate in the final follow-up interview coincided with the outbreak of the COVID-19 global pandemic (Spring 2020). While participant attrition from the time of pre-licensure education to final follow-up may be a perceived limitation in this study, given the longitudinal design, the sample size is still very appropriate for a qualitative, narrative approach (Polkinghorne, 1988). In addition, the impact of the COVID-19 pandemic on healthcare professionals’ desire to engage in research and strains on healthcare systems may have contributed to attrition for the last set of interviews. As a result, the authors acknowledge that the early practice experiences of some healthcare professions may be underrepresented in this study which may be limiting to the findings. The authors confirm however that attentiveness to narrative principles, including thoughtful attention to participant’s narrated experiences, temporality, and setting, have been observed throughout the study to ensure authenticity of participant narratives and the rigor and trustworthiness of the findings.

## Conclusion

Our study findings emphasize that interprofessional socialization and dual identity development for collaborative practice is a process that evolves over time and is contingent on exposure to and working with interprofessional role models and settings and developing new ways of thinking and being in a dual identity. Healthcare professionals’ development of dual identity begins in pre-licensure IPECP but continues to evolve and is shaped by socialization experiences within professional practice settings. There is a need identified in this study to ensure healthcare settings provide the conditions, including time, settings, and contexts that enable practitioners to be change agents for interprofessional and collaborative patient-centered healthcare practice and which contribute positively to practitioners’ development of dual identity. Our study further supports a need for increased continuing IPECP education and lifelong professional development opportunities for health professionals, within healthcare settings and virtual formats, to facilitate accessible socialization opportunities between professions in practice. The exploration of ways that healthcare professionals can continue to learn with, from, and about one another—the very essence of IPECP—within practice settings is essential to the creation of collaborative and effective interprofessional healthcare teams and systems. It is clear that the curricula of health and social care programs should be developed to effect such systems changes.

## Data Availability

The datasets generated and/or analyzed during this study are not publicly available as participants of this study did not give written consent for their data to be shared publicly. Due to the sensitive nature of the research, supporting data is not available.

## Appendix

### List of Abbreviations

CE: Continuing education
COVID-19: Coronavirus disease 2019
IPC: Interprofessional care
IPECP: Interprofessional education for collaborative practice
IPE: Interprofessional education
IPS: Interprofessional socialization
RC: Research coordinator

## References

[bibr1-10497323251333960] American Medical Association . (2021). Bullying in the healthcare workplace: A guide to prevenion and mitigation. https://www.ama-assn.org/system/files/2021-02/workplace-aggression-report.pdf

[bibr2-10497323251333960] Ammentorp J. Chiswell M. Martin P. (2022). Translating knowledge into practice for communication skills training for health care professionals. Patient Education and Counseling, 105(11), 3334–3338. 10.1016/j.pec.2022.08.004 35953393

[bibr3-10497323251333960] Appelbaum N. P. Lockeman K. S. Orr S. Huff T. A. Hogan C. J. Queen B. A. Dow A. W. (2020). Perceived influence of power distance, psychological safety, and team cohesion on team effectiveness. Journal of Interprofessional Care, 34(1), 20–26. 10.1080/13561820.2019.1633290 31381458

[bibr4-10497323251333960] Ateah C. A. Snow W. Wener P. MacDonald L. Metge C. Davis P. Fricke M. Ludwig S. Anderson J. (2011). Stereotyping as a barrier to collaboration: Does interprofessional education make a difference? Nurse Education Today, 31(2), 208–213. 10.1016/j.nedt.2010.06.004 20655633

[bibr5-10497323251333960] Beiter J. (2007). Self-forgiveness: A narrative phenomenological study [Doctoral dissertation]. Duquesne University.

[bibr6-10497323251333960] Brewer M. Flavell H. Jordon J. (2017). Interprofessional team-based placements: The importance of space, place, and facilitation. Journal of Interprofessional Care, 31(4), 429–437. 10.1080/13561820.2017.1308318 28467132

[bibr7-10497323251333960] Canadian Interprofessional Health Collaborative Curricula Committee . (2024). CIHC framework for advancing collaboration. https://cihc-cpis.com/wp-content/uploads/2024/06/CIHC-Competency-Framework.pdf

[bibr8-10497323251333960] Centre for Advancement of Interprofessional Education . (2016). Statement of purpose. https://www.caipe.org/resource/CAIPE-Statement-of-Purpose-2016.pdf

[bibr9-10497323251333960] Chang B. P. Cato K. D. Cassai M. Breen L. (2019). Clinician burnout and its association with team based care in the Emergency Department. The American Journal of Emergency Medicine, 37(11), 2113–2114. 10.1016/j.ajem.2019.06.032 31255426 PMC6917942

[bibr10-10497323251333960] Coggins A. Zaklama R. Szabo R. A. Diaz-Navarro C. Scalese R. J. Krogh K. Eppich W. (2021). Twelve tips for facilitating and implementing clinical debriefing programmes. Medical Teacher, 43(5), 509–517. 10.1080/0142159X.2020.1817349 33032476

[bibr11-10497323251333960] Cruess S. R. Cruess R. L. Steinert Y. (2019). Supporting the development of a professional identity: General principles. Medical Teacher, 41(6), 641–649. 10.1080/0142159X.2018.1536260 30739517

[bibr12-10497323251333960] Grace S. (2021). Models of interprofessional education for healthcare students: A scoping review. Journal of Interprofessional Care, 35(5), 771–783. 10.1080/13561820.2020.1767045 32614628

[bibr13-10497323251333960] Grant A. Kontak J. Jeffers E. Lawson B. MacKenzie A. Burge F. Boulos L. Lackie K. Marshall E. G. Mireault A. Philpott S. Sampalli T. Sheppard-LeMoine D. Martin-Misener R. (2024). Barriers and enablers to implementing interprofessional primary care teams: A narrative review of the literature using the consolidated framework for implementation research. BMC Primary Care, 25(1), Article 25. 10.1186/s12875-023-02240-0 38216867 PMC10785376

[bibr14-10497323251333960] Grissinger M. (2017). Disrespectful behavior in health care: Its impact, why it arises and persists, and how to address it - Part 2. Pharmacy & Therapeutics, 42(2), 74–77. 28163550 PMC5265230

[bibr15-10497323251333960] Gunaldo T. P. Owens J. Andrieu S. C. Mercante D. E. Schiavo J. H. Zorek J. A. (2021). Assessing dental student perceptions after engaging in a longitudinal interprofessional education curriculum: A preliminary study. European Journal of Dental Education, 25(3), 614–620. 10.1111/eje.12639 33269533 PMC8808459

[bibr16-10497323251333960] Guo L. Ryan B. Leditschke I. A. Haines K. J. Cook K. Eriksson L. Olusanya O. Selak T. Shekar K. Ramanan M. (2022). Impact of unacceptable behaviour between healthcare workers on clinical performance and patient outcomes: A systematic review. BMJ Quality and Safety, 31(9), 679–687. 10.1136/bmjqs-2021-013955 35046101

[bibr17-10497323251333960] Hafferty F. O’Donnell J. (2015). The hidden curriculum in health professional education. Dartmouth College Press.

[bibr18-10497323251333960] Jarvis-Selinger S. Pratt D. D. Regehr G. (2012). Competency is not enough: Integrating identity formation into the medical education discourse. Academic Medicine: Journal of the Association of American Medical Colleges, 87(9), 1185–1190. 10.1097/ACM.0b013e3182604968 22836834

[bibr19-10497323251333960] Kavanaugh R. Normington-Gomes H. Graff J. (2023). Professional partners: Longitudinal interprofessional education events with pharmacy and nursing programs. Currents in Pharmacy Teaching and Learning, 15(5), 483–487. 10.1016/j.cptl.2023.04.016 37121868

[bibr20-10497323251333960] Kennie-Kaulbach N. Crespo K. Price S. (2023). A longitudinal narrative case study of interprofessional socialization among pharmacy students. Currents in Pharmacy Teaching and Learning, 15(11), 925–932. 10.1016/j.cptl.2023.09.001 37718221

[bibr21-10497323251333960] Khalili H. Lackie K. Langlois S. Wetzlmair L. C. (2022). Global IPE situational analysis results final report. Interprofessional Research Global. https://interprofessionalresearch.global/wp-content/uploads/2022/10/Global-IPE-Situational-Analysis-Final-Report-October-2022-1.pdf 10.1080/13561820.2023.2287023 38126193

[bibr22-10497323251333960] Khalili H. Lising D. Gilbert J. Thistlewaite J. Pfeifle A. Maxwell B. Başer Kolcu I. Langlois S. Najjar G. MacMillan K. M. Al-Hamdan Z. Schneider C. Kolu G. El-Awaisi A. Ward H. Rodrigues F. J. (2021). Building resilience in health care in the time of COVID-19 through collaboration- A call to action. Interprofessional Research Global. https://www.interprofessionalresearch.global

[bibr23-10497323251333960] Khalili H. Orchard C. (2020). The effects of an IPS-based IPE program on interprofessional socialization and dual identity development. Journal of Interprofessional Care, 1–11. 10.1080/13561820.2019.1709427 32019374

[bibr24-10497323251333960] Khalili H. Orchard C. Laschinger H. K. S. Farah R. (2013). An interprofessional socialization framework for developing an interprofessional identity among health professions students. Journal of Interprofessional Care, 27(6), 448–453. 10.3109/13561820.2013.804042 23777592

[bibr25-10497323251333960] Khalili H. Pandey J. Langlois S. Park V. Brown R. El-Awaisi A. MacMillan K. M. Daulton B. Green C. Konrad S. C. Kolcu G. Başer Kolcu M. I. Baugh G. Pfeifle A. Wetzlmair L. C. Breitbach A. (2024). Forward thinking and adaptability to sustain and advance IPECP in healthcare transformation following the COVID-19 pandemic. The Internet Journal of Allied Health Sciences and Practice, 22(1), Article 18. https://nsuworks.nova.edu/ijahsp/vol22/iss1/18/

[bibr26-10497323251333960] Khalili H. Price S. (2022). From uniprofessionality to interprofessionality: Dual vs dueling identities in healthcare. Journal of Interprofessional Care, 36(3), 473–478. 10.1080/13561820.2021.1928029 34139953

[bibr28-10497323251333960] Kruijver I. Kerkstra A. Francke A. Bensing J. van de Wiel H. (2000). Evaluation of communication training programs in nursing care: A review of the literature. Patient Education and Counseling, 39(1), 129–145. 10.1016/S0738-3991(99)00096-8 11013554

[bibr29-10497323251333960] Maben J. Aunger J. A. Abrams R. Wright J. M. Pearson M. Westbrook J. I. Jones A. Mannion R. (2023). Interventions to address unprofessional behaviours between staff in acute care: What works for whom and why? A realist review. BMC Medicine, 21(1), Article 403. 10.1186/s12916-023-03102-3 37904186 PMC10617100

[bibr30-10497323251333960] Mukhalalati B. Aly A. Yakti O. Elshami S. Daud A. Awaisu A. Sethi A. El-Awaisi A. Stewart D. Abu-Hijleh M. F. Austin Z. (2024). Examining the perception of undergraduate health professional students of their learning environment, learning experience and professional identity development: A mixed-methods study. BMC Medical Education, 24(1), Article 886. 10.1186/s12909-024-05875-4 39152424 PMC11330008

[bibr31-10497323251333960] Noyes A. (2022). Navigating the hierarchy: Communicating power relationships in collaborative health care groups. Management Communication Quarterly, 36(1), 62–91. 10.1177/08933189211025737

[bibr32-10497323251333960] Odole A. C. Odunaiya N. A. Ajadi O. I. (2019). Interprofessional education among Nigerian clinical students: Implications for interprofessional care. Journal of Interprofessional Care, 33(6), 645–653. 10.1080/13561820.2018.1544545 30428727

[bibr33-10497323251333960] Pettigrew T. (1998). Intergroup contact theory. Annual Review of Psychology, 49(1), 65–85. 10.1146/annurev.psych.49.1.65 15012467

[bibr34-10497323251333960] Polkinghorne D. (1988). Narrative knowing and the human science. State University of New York Press.

[bibr35-10497323251333960] Polkinghorne D. (1995). Narrative configuration in qualitative analysis. International Journal of Qualitative Studies in Education, 8(1), 5–23. 10.1080/0951839950080103

[bibr36-10497323251333960] Polkinghorne D. (1997). Phenomenology and narrative psychology. Duquesne University, The Simon Silverman Phenomenology Centre.

[bibr37-10497323251333960] Price S. Van Dam L. Sim M. Andrews C. Gilbert J. H. V. Lackie K. Almost J. Kennie-Kaulbach N. Sutton E. Khalili H. (2024). A longitudinal study of interprofessional education experiences among health professional graduates. Advances in Health Sciences Education. 10.1007/s10459-024-10374-8 39316361

[bibr38-10497323251333960] Price S. L. Sim M. Little V. Almost J. Andrews C. Davies H. Harman K. Khalili H. Reeves S. Sutton E. LeBrun J. (2021a). Pre-entry perceptions of students entering five health professions: Implications for interprofessional education and collaboration. Journal of Interprofessional Care, 35(1), 83–91. 10.1080/13561820.2019.1702514 31865829

[bibr39-10497323251333960] Price S. L. Sim S. M. Little V. Almost J. Andrews C. Davies H. Harman K. Khalili H. Sutton E. LeBrun J. (2021b). A longitudinal, narrative study of professional socialisation among health students. Medical Education, 55(4), 478–485. 10.1111/medu.14437 33332659

[bibr40-10497323251333960] Reeves S. Perrier L. Goldman J. Freeth D. Zwarenstein M. (2013). Interprofessional education: Effects on professional practice and healthcare outcomes (update). Cochrane Database of Systematic Reviews, 2013(3), Article CD002213. 10.1002/14651858.CD002213.pub3 23543515 PMC6513239

[bibr41-10497323251333960] Reinders J. Krijnen W. P. (2023). Interprofessional identity and motivation towards interprofessional collaboration. Medical Education, 57(11), 1068–1078. 10.1111/medu.15096 37073763

[bibr42-10497323251333960] Reinders J. J. Krijnen W. P. Goldschmidt A. M. van Offenbeek M. A. G. Stegenga B. van der Schans C. P. (2018). Changing dominance in mixed profession groups: Putting theory into practice. European Journal of Work and Organizational Psychology, 27(3), 375–386. 10.1080/1359432X.2018.1458712

[bibr100-10497323251333960] Reinders J. Lycklama A. N. M. Van Der Schans C. P. Krijnen W. P. (2020). The development and psychometric evaluvation of an interprofessional identity measure: Extended Professional Identity Scale (EPIS). Journal of Interprofessional Care, 1–13. 10.1080/13561820.2020.1713064 32013632

[bibr43-10497323251333960] Ricoeur P. (1991). From text to action: Essays in hermeneutics II. Athlone Press.

[bibr44-10497323251333960] Ricoeur P. (1997). A response by Paul Ricoeur. Paul Ricouer and narrative: Context and contestation. University of Alberta.

[bibr45-10497323251333960] Sadeghi Avval Shahr H. Yazdani S. Afshar L. (2019). Professional socialization: An analytical definition. Journal of Medical Ethics and History of Medicine, 12(17), 1–14. 10.18502/jmehm.v12i17.2016 32328230 PMC7166248

[bibr46-10497323251333960] Shelvey B. M. Coulman S. A. John D. N. (2016). Evaluating an undergraduate interprofessional education session for medical and pharmacy undergraduates on therapeutics and prescribing: The medical student perspective. Advances in Medical Education and Practice, 7, 661–670. 10.2147/AMEP.S116618 27980450 PMC5147417

[bibr47-10497323251333960] Stets J. E. Burke P. J. (2000). Identity theory and social identity theory. Social Psychology Quarterly, 63(3), Article 224. 10.2307/2695870

[bibr48-10497323251333960] Stull C. Lei F. North S. (2024). First-year health professions students’ interprofessional identity development following participation in a brief introductory interprofessional activity: A qualitative study. Journal of Interprofessional Care, 1–10. 10.1080/13561820.2024.2391353 39199006

[bibr49-10497323251333960] Tajfel H. Turner J. (1979) An integrative theory of intergroup conflict. In Austin W. G. Worchel S. (Eds.), The social psychology of intergroup relations (pp. 33–37). Brooks/Cole.

[bibr50-10497323251333960] Tong A. Sainsbury P. Craig J. (2007). Consolidated criteria for reporting qualitative research (COREQ): A 32-item checklist for interviews and focus groups. International Journal for Quality in Health Care, 19(6), 349–357. 10.1093/intqhc/mzm042 17872937

[bibr51-10497323251333960] Tong R. Brewer M. Flavell H. Roberts L. (2022). Exploring interprofessional identity development in healthcare graduates and its impact on practice. PLoS One, 17(5), Article e0268745. 10.1371/journal.pone.0268745 35622839 PMC9140281

[bibr52-10497323251333960] Van Dam L. Sim M. Sutton E. Price S. (2024). ‘It takes a village to raise a resident’: Lessons learned on interprofessional socialization and collaborative practice from recent medical graduates. Medical Science Educator. 1–11. 10.1007/s40670-024-02247-x PMC12058583 40353031

[bibr53-10497323251333960] Velásquez S. T. Ferguson D. Lemke K. C. Bland L. Ajtai R. Amezaga B. Cleveland J. Ford L. A. Lopez E. Richardson W. Saenz D. Zorek J. A. (2022). Interprofessional communication in medical simulation: Findings from a scoping review and implications for academic medicine. BMC Medical Education, 22(1), Article 204. 10.1186/s12909-022-03226-9 35346159 PMC8962252

[bibr54-10497323251333960] Welp A. Meier L. Manser T. (2016). The interplay between teamwork, clinicians’ emotional exhaustion, and clinician-rated patient safety: A longitudinal study. Critical Care, 20(1), Article 110. 10.1186/s13054-016-1282-9 27095501 PMC4837537

[bibr55-10497323251333960] Wenger-Trayner E. Wenger-Trayner B. (2015). An introduction to communities of practice: A brief overview of the concept and its uses. https://www.wenger-trayner.com/wp-content/uploads/2022/06/15-06-Brief-introduction-to-communities-of-practice.pdf

[bibr56-10497323251333960] Whiting L. Caldwell C. Akers E. (2016). An examination of interprofessional education in a pre-registration children’s nursing course. Nursing Children and Young People, 28(6), 22–27. 10.7748/ncyp.2016.e747 27387633

[bibr57-10497323251333960] Wolderslund M. Kofoed P.-E. Ammentorp J. (2021). The effectiveness of a person-centred communication skills training programme for the health care professionals of a large hospital in Denmark. Patient Education and Counseling, 104(6), 1423–1430. 10.1016/j.pec.2020.11.018 33303282

[bibr58-10497323251333960] World Health Organization . (2010). Framework for action on interprofessional education & collaborative practice. https://www.who.int/publications/i/item/framework-for-action-on-interprofessional-education-collaborative-practice 21174039

